# The complete mitochondrial genome of the breed Ningbo brown-marbled grouper (*Epinephelus fuscoguttatus*)

**DOI:** 10.1080/23802359.2019.1623108

**Published:** 2019-07-10

**Authors:** Gang Liu, Zhizhong Gong, Qingyue Li

**Affiliations:** School of Life Sciences, Anhui Medical University, Hefei, P. R. China

**Keywords:** *Epinephelus fuscoguttatus*, NGS technologies, complete mitochondrial genome sequences

## Abstract

We used NGS technologies to resequence the complete mitochondrial genome of (*Epinephelus fuscoguttatus*). The complete mitochondrial genome of the breed Ningbo brown-marbled grouper is a 16,373 bp circular molecule, which contains 37 typical mitochondrial genes (13 protein-coding genes, two rRNAs, and 22 tRNAs) and a D-loop. Its gene arrangement pattern is the same as the accession no. KP013758. Comparing to accession no. KP013758 sequence, the D-loop region is much shorter. ATG is the most common start codon, and TAA is the most common stop codons; the 12S rRNA and 16S rRNA are located between the tRNA^Phe^ and tRNA^Leu^ genes and are separated by the tRNA^Val^ gene. Phylogenetic trees indicated *E. fuscoguttatus* has close relative with *Epinephelus coioides* and *Epinephelus malabaricus.*

*Epinephelus fuscoguttatus* is known as the brown-marbled grouper, belongs to order Perciformes, family Serranidae, which mainly distributes in Indo-Pacific from eastern Africa to the Phoenix Islands, including Red Sea, China, Japan, and Australia (Peng et al. 2016). The species is a high market value and serves as an important commercial mariculture fish species, due to its excellent food quality, abundant nutrients and rapid growth, which has been widely distributed in South China (Zhu et al. 2016). In this study, we sequenced and characterized the complete mitochondrial genome of breed Ningbo brown-marbled brouper used NGS technologies and expected that it could provide more molecular data for the genetic studies of the breed population.

The muscle sample of breed brown-marbled grouper was collected from a fish farm in Ningbo City of Zhejiang Province, China. The sample was stored at −80 °C in the Cancer Cell Biology Laboratory, School of Life Sciences, Anhui Medical University (Sample codes are AHMUFish-20181023). The mtDNA was sequenced by Genesky Biotech Co., Ltd. (Shanghai, China) using NGS technologies. The complete mtDNA sequence of breed Ningbo brown-marbled grouper has been assigned with GenBank accession number MK791189. The length of the complete mtDNA sequence is 16,373 bp, shorter than the species which has been published in 2016 (accession no. KP013758) (Peng et al. 2016). The new mtDNA consisting of 13 protein-coding genes, two ribosomal RNA genes (12S rRNA and 16S rRNA), 22 transfer RNA genes (tRNA), and one control region, which is the same as the accession no. KP013758 and other fish (Wang et al. 2008; Peng et al. 2016). Comparing to accession no. KP013758 sequence, the D-loop region is much shorter. All the protein-coding genes begin with an ATG start codon except for COX1 started with GTG and ATP6 started with CTG, and TAA is the most common stop codons. 12S rRNA and 16S rRNA are located between the tRNA^Phe^ and tRNA^Leu^ and are separated by the tRNA^Val^ with the same situation found in the accession no. KP013758 (Peng et al. 2016).

Phylogenetic trees were estimated using ML and BI methods, based on the complete mtDNA of six *Epinephelus* species, and corresponding *Variola louti* (NC_022138) sequence was used as an outgroup, sharing similar topologies and high node support values (Figure 1). *Epinephelus fuscoguttatus* has close relative with *Epinephelus coioides* and *Epinephelus malabaricus.*

**Figure 1.**
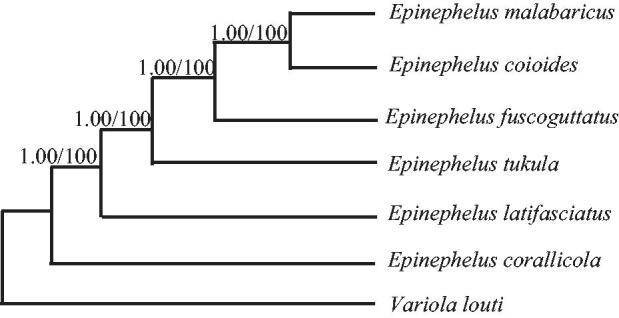
Phylogenetic relationships among the six *Epinephelus* species based on complete mtDNA sequences. Numbers at each node are Bayesian posterior probabilities (left) and maximum-likelihood bootstrap proportions (estimated from 100 pseudoreplicates) (right). The accession number in GenBank in this study: *Variola louti* (NC_022138), *Epinephelus corallicola* (KP072053), *Epinephelus malabaricus* (KM873711), *Epinephelus tukula* (KJ414470), *Epinephelus coioides* (KM377093), and *Epinephelus latifasciatus* (KC480177).

## Nucleotide sequence accession number

The complete mtDNA sequence of Breed Ningbo brown-marbled grouper (*E. fuscoguttatus*) has been assigned with GenBank accession number MK791189.
